# Occupational Safety Analysis for COVID-Instigated Repurposed Manufacturing Lines: Use of Nanomaterials in Injection Moulding

**DOI:** 10.3390/polym14122418

**Published:** 2022-06-14

**Authors:** Spyridon Damilos, Stratos Saliakas, Ioannis Kokkinopoulos, Panagiotis Karayannis, Melpo Karamitrou, Aikaterini-Flora Trompeta, Costas Charitidis, Elias P. Koumoulos

**Affiliations:** 1 Innovation in Research & Engineering Solutions (IRES), 1780 Wemmel, Belgium; sdamilos@innovation-res.eu (S.D.); esaliakas@innovation-res.eu (S.S.); jkokkinopoulos@innovation-res.eu (I.K.); karayannisp@innovation-res.eu (P.K.); 2 Research Lab of Advanced, Composites, Nanomaterials and Nanotechnology (R-NanoLab), School of Chemical Engineering, National Technical University of Athens, Zographos, 15780 Athens, Greece; mkaramitru@chemeng.ntua.gr (M.K.); ktrompeta@chemeng.ntua.gr (A.-F.T.); charitidis@chemeng.ntua.gr (C.C.)

**Keywords:** injection moulding, nanocomposites, repurposing, nanomaterials, failure mode and effect analysis, risk analysis, nanosafety

## Abstract

The COVID-19 pandemic instigated massive production of critical medical supplies and personal protective equipment. Injection moulding (IM) is considered the most prominent thermoplastic part manufacturing technique, offering the use of a large variety of feedstocks and rapid production capacity. Within the context of the European Commission-funded imPURE project, the benefits of IM have been exploited in repurposed IM lines to accommodate the use of nanocomposites and introduce the unique properties of nanomaterials. However, these amendments in the manufacturing lines highlighted the need for targeted and thorough occupational risk analysis due to the potential exposure of workers to airborne nanomaterials and fumes, as well as the introduction of additional occupational hazards. In this work, a safety-oriented failure mode and effects analysis (FMEA) was implemented to evaluate the main hazards in repurposed IM lines using acrylonitrile butadiene styrene (ABS) matrix and silver nanoparticles (AgNPs) as additives. Twenty-eight failure modes were identified, with the upper quartile including the seven failure modes presenting the highest risk priority numbers (*RPN*), signifying a need for immediate control action. Additionally, a nanosafety control-banding tool allowed hazard classification and the identification of control actions required for mitigation of occupation risks due to the released airborne silver nanoparticles.

## 1. Introduction

Injection moulding (IM) is one of the most common manufacturing techniques because of the large variety of different feedstocks, including polymers, metals, ceramics and composites, it allows, as well as the low production costs it entails [1]. There has been continuous optimization for the processing of thermoplastic polymers [2,3], while recent advancements have led to the manufacturing of nanocomposite parts and products [4]. Among the species of nanomaterials that have been used for additives in IM manufacturing case studies are multiwalled carbon nanotubes (MWCNTs) in polypropylene (PP) nanocomposites for tuning electrical properties [5] and TiO_2_ nanoparticles in high-density polyethylene (HDPE) nanocomposites for modifying thermal and mechanical properties [6]. Other additives that are commonly used in IM are short carbon or glass fibres, with the aim to improve the part’s mechanical properties [7]. The preparation of the nanocomposite masterbatch input material is commonly performed through twin-screw extrusion, and while the processing parameters required to accommodate the additive nanomaterials or fibres have been extensively discussed [4,5,6,7], supportive elements of the process that may also need adjustment have not emphasized. These include the possible impact of the additives on the process’ emission potential as well as nanosafety elements, which affect the occupational safety aspects of manufacturing. These aspects were the focal points of the present work.

In a recent review study, Hassan et al., discussed the types, characteristics, and potential applications of various nanocomposite materials [8]. The introduction of enhanced electrical, mechanical, and optical properties, along with the development of unique properties such as multifunctionality, based on the species of nanomaterial used as additive, was expressed as a definitively beneficial element of nanocomposite materials. Among various highly promising areas for application of nanocomposites described were coatings, automobile and aerospace applications, and electronics. The authors reported the application of silver nanoparticles in several cases aiming to establish antimicrobial properties as well as in biomedical applications.

Hilliou and Covas performed a review of the production and processing routes for the development of polymer-based nanocomposites [9]. A predominant challenge discussed by the authors was the achievement of proper nanoparticle dispersion into the matrix through understanding of the dispersion mechanisms. Injection moulding was discussed as a primary technique for nanocomposite part development. The basic principles for optimization of the IM-produced part’s properties were presented, including definition of the optimal temperature settings to avoid polymer thermal degradation from excessively high temperatures as well as the weak regions in the part, caused by inefficient melting, that result from low temperatures. Process-specific defects such as warping were identified and presented significant connections with the temperature setup. Although various technological challenges and aspects were considered in these works, a dedicated focus on nanosafety aspects and possible releases of nanomaterials within the scope of the processes has not been allocated. More specifically, modification of the occupational safety elements of nanocomposite activities compared with processing of a conventional polymer has not been intensively evaluated in the literature.

Within the scope of nanosafety, Froggett et al., reviewed the mechanisms and patterns of releases from solid nanocomposites throughout different occupational and consumer-related activities [10]. Based on the studies evaluated, the authors highlighted that the release occurrences could be grouped into classes corresponding to the activity taking place. These groups were “machining, weathering, washing, contact and incineration”. Various types of release forms were identified, the most frequently occurring of which were fragments/particles of the matrix and matrix particles with embedded nanomaterials. The thermal processing of nanocomposites, which occurs during manufacturing activities such as compounding or injection moulding, has not been extensively evaluated as an exposure scenario. Therefore, there exist knowledge gaps related to possible releases during these processes. Evaluation and addressing of these safety aspects would support widespread adoption of highly promising nanocomposite materials.

The COVID-19 pandemic and its expeditious spread on a worldwide scale within the first quarter of 2020 led to urgent increases in demand for medical products and equipment, such as hand sanitizers, personal respirator masks, and ventilators, highlighting substantial shortages in many cases [11]. As a response to these extraordinary conditions, a wide variety of companies from many different industrial sectors managed to rapidly repurpose their processes and product design to provide support in the COVID-19 situation by producing medical-related products [12]. Medical plastics are a common application of the injection moulding manufacturing technique [13], and thus, IM showed compatibility for use in the COVID-19 response. Among various medical applications, Tay et al., demonstrated a case study for a novel COVID-19 nasopharyngeal swab manufacturing technique through IM using biocompatible nylon material. The authors reported that the process’s high throughput, scale-up potential, and cost-effectiveness benefits could be exploited to produce a clinically safe product [14]. Oladapo et al., reviewed the potential of 3D printing technologies, such as fused filament fabrication (FFF) implementing various biomaterial-based feedstocks, as tools for the manufacturing of respirators and other medicals devices in the fight against COVID-19, and concluded that the benefits of this technology outweighed the intrinsic production and ethical risks [15]. In comparison with IM, FFF offers more capabilities in terms of produced shape complexity and the prospect of personalized products through easy customization of the design. However, higher manufacturing time has been described as a substantial shortcoming of FFF compared with IM, along with lower mechanical strength (particularly in the item’s Z axis) and quality and print defect issues [16]. The quick manufacturing capacity offered by IM is a particularly important element to consider when designing a repurposing scheme, since the rapid production of items to meet rising demand will most likely be one of the key objectives of the endeavour.

The technical part of the repurposing process involves an array of challenges, since modifications in several parts of the product value chain are required. Kapoor et al., attempted to review these barriers, including hindrances faced in the general manufacturing industry, such as skill gaps of the manufacturing workforce and the financial requirements to implement new technologies, along with barriers introduced by COVID-19, such as the disruption of the production networks and supply chains due to lockdowns and potential shortages in raw materials [17]. Issues related to safety have been a part of the discussion on repurposing challenges. However, the discussion has to date focused mainly on product clinical safety and sterility or the impact of COVID-19 safety protocols on updated manufacturing requirements (e.g., social distancing in manufacturing facilities) [17]; additional, case-specific safety concerns should be taken into account in repurposed manufacturing lines related to the operation protocols and equipment used. Poduval et al., identified further obstacles, such as regulatory barriers (e.g., license to manufacture a specific product, environmental aspects), concerns over the quality of the products of the repurposed line being equivalent to that of commercial products, and innovation barriers related to identifying the exact method to repurpose the manufacturing lines in the most efficient and minimally disruptive way. Nevertheless, safety-related barriers were out of the scope of their study [18].

The safety aspects of industrial-scale injection moulding have been thoroughly explored and documented in recent standards, e.g., from the International Organization for Standardization (ISO). ISO 20430:2020 [19] presented a general overview of the safety measures to be used towards the minimization of risk during injection moulding activities. The implementation of guards enforcing safe distances was further detailed in ISO 13857:2019 [20] on machinery safety. In order to enhance the operators’ risk awareness, hazard zones should be marked with the relevant warning signs included in ISO 7010:2019 [21]. National safety organizations have also created tools in order to educate and guide the operators. The Health Service Executive (HSE) published an information sheet with a series of safety checks that can be performed by operators to minimize risk in injection moulding facilities [22]. Acknowledging high rates of injuries, the Occupational Safety and Health Administration (OSHA) developed a machine guarding e-Tool, which provides a visual safety tour for horizontal injection moulding machines [23]. These documents focused on hazards arising from the use moving machinery parts and the handling of heavy tools in large-scale production lines.

It is worth noting that the aforementioned standards focused on the occupational health and safety risks around injection moulding without taking into account the safety concern resulting from airborne particulate emissions and nano-specific hazards from the addition of nanomaterials (NMs) into injection moulding manufacturing lines. Thermal, noise, and trip hazards have also been briefly mentioned. On a pilot-line scale, many of the controls described are hard to implement or completely inapplicable, mainly because of prohibitive costs and/or a low level of process automation. Occupational health and safety aspects of repurposed processes and production lines have not been extensively discussed, although these issues may significantly affect the long-term viability of the repurposing. The production alteration process may introduce new materials (including in nanoscale), pre- and postprocessing steps, or modifications in the process workflow and setup, which may raise considerable challenges to the established safety system.

Nanomaterials include engineered (ENMs) and incidental materials of which at least one dimension is in the nanoscale region (1–100 nm), presenting unique properties in comparison with their bulk alternatives due to their higher surface area relative to their mass leading to increased chemical reactivity, different patterns of absorption into the body, and different health effects caused [24]. Recent studies have outlined the health-related hazards due to nanomaterial exposure [25]. The basic routes of exposure are respiratory, oral, and skin, but there is also a risk of potential translocation of nanomaterial to vital organs leading to adverse health effects [26]. Airborne particulate matter affects different areas of the respiratory tract (nasal, tracheobronchial, and alveolar areas) based on their diameter size [27]; although microscale particles can be easily cleared from the nasal cavity, NMs tend to deposit in the alveolar region, leading to inflammation [27]. Literature studies have investigated the toxicological effects of NMs, showing that on the cellular level, these materials can cause the production of reactive oxygen species (ROS) and oxidative stress, with subsequent mitochondrial dysfunction, deoxyribonucleic acid (DNA) and protein damage, apoptosis, etc. leading to cytotoxic and mutagenic effects [28]. Medici et al. [29] further reviewed the parameters affecting the toxicity potential of several metal nanomaterials, showcasing that these toxicological effects depended on several parameters, such as chemical composition, surface charge, morphology, and shape. Analysing different antimicrobial nanomaterial types (silver, copper oxide, and zinc oxide nanoparticles), Bondarenko et al., also discussed the effect of particulate concentration on the toxicological potential and toxicity of both the NMs and their released ions [30].

Studies have explored health hazards during injection moulding of nanocomposites, showcasing the associated risks from emitted particulate matter not only during the injection moulding of the plastic but during the secondary, supplementary processes (e.g., sieving, milling, etc.) [31]. Studies associating hazards and risks from airborne NMs are still limited regarding different material feedstocks (including the polymer matrices and nanoadditives), while the occupational exposure limits do not include the overall spectrum of inhalable and respirable particulate matter [31]. The mechanisms of material release from nanocomposites were presented in detail in ISO/TR 22293:2021 [32]. On the other hand, in the methodology developed by Lithner et al. [33], the hazards of the polymer matrix can be evaluated separately from the ENMs based on their monomers. Handling of the produced nanocomposites is the primary means of contact with ENMs across the life stages of the nanoenabled products. Pelclova et al. [34] analysed aerosol samples from on-site measurements during the injection moulding of thermoplastic and thermosetting nanocomposite materials, focusing only on the toxicological analysis of the collected airborne particulate matter, and showed that emissions during nanocomposite processing could potentially lead to respiratory inflammation, indicating a need for exposure controls to mitigate the associated risks. Similarly, Boonruksa et al. [35] studied the release of carbon nanotubes (CNTs) during the processing (loading, melting, moulding, grinding) and recycling of CNT polypropylene composites (CNT–PP), where they confirmed the release of PP nanoparticles as well as PP particles with embedded CNTs. These works presented toxicological and exposure concerns. However, potential mitigating actions to minimize the release and the associate risks of PP particles with or without embedded CNTs were not defined. Hence, nanomaterial or nanoenabled polymer release may take place because of mechanical stress (e.g., sawing, shredding) and intense thermal stress during incineration [32], emphasizing the importance of increased concern during nanocomposites’ life cycles.

During the pandemic situation and because of shortages in raw materials, alternatives should be investigated, especially when repurposing of an industrial production line is vital for the production of critical medical supplies. Within the Horizon 2020 imPURE project (Grant Agreement 101016262) [36], bulk modification strategies for the incorporation of low-concentration nanoparticles for new functionalities of existing polymers have been tried out to offer added value to the products thereby developed. The concept behind this process is the exploitation of polymers available in the IM industry and the upgrading of their properties and functionality to make them more compatible for use in medical applications. The objective is to use these materials as supportive materials alongside medical-grade polymers, which are used exclusively for the production of medical devices and cannot be substituted, thus giving an opportunity to react fast when a crisis interrupts the supply chain of raw materials. In this case, if easily accessible nanomaterials were available (such as silver, zinc, and copper nanoparticles), they could be incorporated in the existing polymers by preparing nanocompounds and be used for the production of medical supplies with antimicrobial properties, offering an advanced characteristic to the final product (removal of pathogens).

In the present study, nanomaterials were introduced as additives in the feedstock polymer within repurposed IM lines in order to meet the end-product goals set for the imPURE project. The development of effective antimicrobial polymers for medical devices seems increasingly important because of the extensive use of polymers in the medical sector [37]. Unlike other antimicrobial agents, metal/metal oxides are stable under conditions currently found in the industry, allowing their use as additives [38]. Previous investigations have used copper nanocomposites to enhance the antimicrobial properties of polymers used in injection moulding and additive manufacturing to develop medical devices [39]. To the best of our knowledge, silver nanoparticles (AgNPs) in combination with acrylonitrile butadiene styrene (ABS) have not been used in IM applications for the manufacturing of medical devices (i.e., oximeters). Thus, it is important to perform an occupational safety analysis, especially for an industrial process that includes nanoparticles. The occupational risk assessment and management of nanoparticles often requires a dedicated, nano-specific methodology [25], which is a direct challenge to the administration and workforce to apply. This is due to the fact that toxicological insights on the potential hazard identity of the nanomaterial in question are required to understand the health risks. Understanding and evaluating the prospect of occupational exposure to nanomaterials also requires a specific set of tools and expertise. Additionally, the part of nano-risk perception [40] is critical, since the workforce may be unaware of nanomaterial hazards or have an unclear awareness of the magnitude of the hazards. To address this challenge, a combination of a conventional (Failure Mode and Effects Analysis) and a nano-specific (Stoffenmanager Nano) risk assessment approach to assess risks related to the repurposed manufacturing line is presented in this study.

The occupational risk assessment of a repurposed production line was the focal piece of this work, with the objective to highlight this relatively unexplored aspect of the repurposing process. In this way, this work aimed to showcase how similar rapid adaptation concepts that are required from the technical part of the repurposing can be applied by support staff, such as occupational safety technicians, to enhance the overall viability and sustainability of the repurposing process. Given that safety concerns of this nature can be a deterring factor for the extensive adaptation of repurposed manufacturing concepts, this study could be valuable for workplaces investigating the prospect of applying repurposing adjustments in their production processes. It was within the scope of the present work to describe and implement a methodology for assessing the newly introduced risks and identifying safety controls needed. This concept is also closely connected to assessing the economic investment requirements of a repurposing approach, which, as discussed above, can be an additional disincentive for repurposing a manufacturing line. The risk assessment process could assist in the identification of the safety assets that must be updated in the workplace and in clearly defining the economic investments needed in terms of reinforcing occupational safety.

## 2. Materials and Methods

### 2.1. Materials

Among various nanomaterials, silver nanoparticles have been among the most popular objects of study in recent decades. Because of their large surface-to-volume ratio, silver nanoparticles exhibit remarkable antibacterial activity, even at a low concentration [41]. In this study, commercial spherical AgNPs of 18 nm size with ~0.25% poly-vinylpyrrolidone (PVP) capping ligand (purity: >99.995%) from Nanografi Nano Technology (Ankara, Turkey) were used (in powder form) for the preparation of antimicrobial thermoplastic nanocomposites. ABS provided by Trinseo PLC (Berwyn, PA, USA) was selected as the polymer matrix for this study. The selected grade, Magnum 3453 Natural, is a general-purpose injection moulding resin suitable for a wide range of applications. The product combined a medium-to-high impact performance with good flowability for the injection moulding process.

### 2.2. Process for Preparation of Nanocomposite Materials

**Figure 1:**
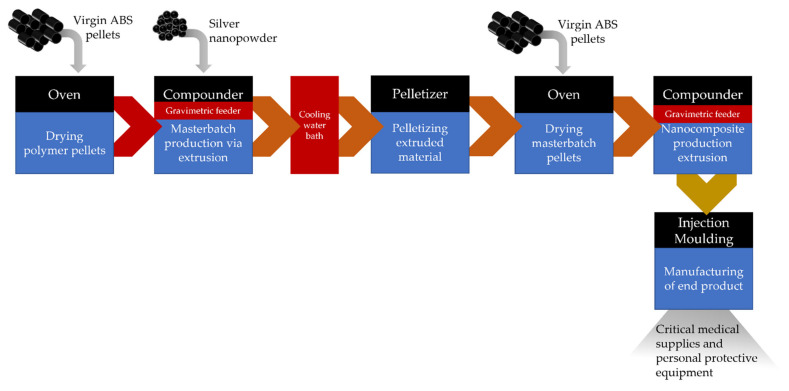
Process flow diagram of the manufacturing pilot line.

**Figure 2:**
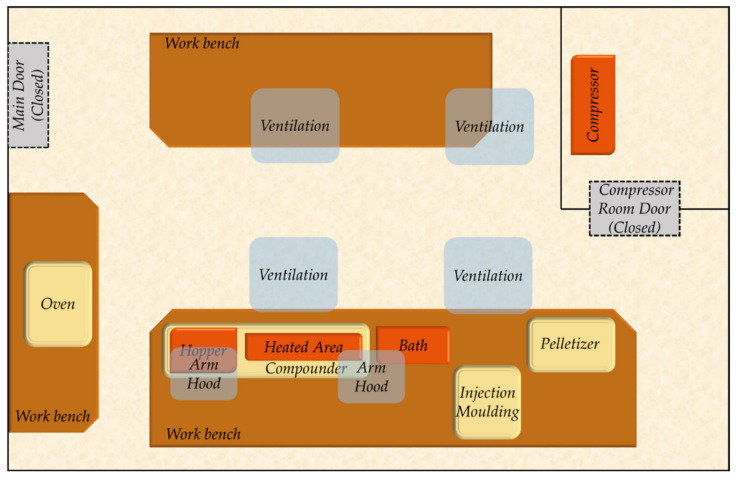
Top-down illustration of the manufacturing pilot line.

The core process for preparing the nanocomposite material with antimicrobial properties was compounding through a twin screw extruder, which enables the incorporation of the AgNPs in the ABS in a melt form (Figure 1). Initially, the thermoplastic material, in the form of pellets, was dried in an oven at 80 °C for 4 h as indicated by the Technical Data Sheet. For the compounding process of the ABS with Ag nanopowder, a Thermo Scientific™ Process 11 Parallel corotating Twin-Screw Extruder (Thermofisher Scientific, Karlsruhe, Germany) with a barrel diameter 11 mm, barrel length-to-diameter (L/D) ratio 40 L/D, and 7 × 5 L/D electrical heated zones was used. The compounder, apart from the main hopper, was equipped with a twin screw Brabender MT-S side gravimetric feeder for nanopowder feeding in dry form. Twin-screw rotation speed was set at 400 rpm, and the 7 heating zones of the temperature profile were 120 °C,180 °C, 200 °C, 220 °C, 220 °C, 220 °C, 220 °C. The process included the initial melting of the polymer, the addition and dispersion of the nanopowder in the melt in the highest possible concentration (masterbatch preparation), the extrusion of the nanocomposite in a rod form, the stabilization of the nanocomposite through water cooling, and finally the cutting of the material into pellet form to be used as feedstock material. The produced nanocomposite pellets were then dried in the oven at 80 °C for 4 h before they were reintroduced into the compounder. Masterbatch pellets were combined with virgin ABS during compounding in order to adjust the concentration of AgNPs in the final material. The extruded composite was then transferred to the injection moulding system (Xplore, micro-injection moulder, Sittard, The Netherlands), and the process parameters were set properly. The micro-injection moulder consisted of a temperature-controlled mould, set at 70 °C, housing a conically shaped mould and a heated, removable injection nozzle unit set at 220 °C. The fitting of the divisible, conically shaped mould into the mould housing prevented the flashing of material. For the preparation of plastic parts/components, a mould with a specific cavity was selected depending on the desired geometry. The IM barrel was filled with a direct connection to the compounder’s die. Afterwards, the nanocomposite material was injected into the temperature-controlled mould with a plunger powered by compressed air. Three steps followed injection; holding pressure and time were controlled at 7 bar for 7 s, 9 bar for 0.1 s, and 9 bar for 10 s. The mould was then removed from the machine and opened by hand. Figure 2 presents an illustration of the whole process.

The manufacturing line involved four main nodes: the compounder, the injection moulding system, the pelletizer, and the oven. These involved their own sets of distinct hazards, while some types of hazards may have recurred (e.g., high temperatures). It was crucial to apply a methodology that combined hazard identification, risk-based prioritization, and definition of appropriate controls in order to gain a robust understanding of the occupational risks involved.

### 2.3. Failure Mode and Effect Analysis (FMEA)

Failure mode and effect analysis (FMEA) methodology is a step-by-step process in which risk is associated with the identified failure modes of each process step or piece of equipment, the corresponding effects and causes are identified, and issues for corrective action are prioritized based on the failure severity, occurrence and detectability, as described in SAE J 1739-2009 Standard [42]. The methodology presented in the Standard was tailored for the detection and characterization of failure modes that could lead to the disruption of a manufacturing process [42]. A safety-oriented FMEA based on the same principles has been presented in the literature, with the authors analysing occupational safety during civil construction and maintenance work [43,44,45]. A similar safety approach, refined for the assessment of the repurposed injection moulding line, was applied in the current study.

The risk was calculated based on three elements identified per failure mode, as shown in Table 1 (the values assigned for each level are shown below):

- Severity (*S*)—ranked from 1 to 5; associated with the most serious effect for a given failure mode (1: least serious, 5: most serious).
- Occurrence (*O*)—ranked from 1 to 5; associated with the likelihood that the failure mode cause will be present in the item being analysed (1: unlikely, 5: most likely).
- Detection (*D*)—ranked from 1 to 5; associated with the likelihood that the failure mode can be detected and prevented based on the current process controls (1: most likely to be detected, 5: most unlikely to be detected).

The risk associated with a failure mode and associated cause was calculated based on these three elements:

(1) RPN=S×O×D

The risk priority number (*RPN*) is a numerical ranking of the risk of each potential failure mode/cause. The higher the *RPN* was, the higher the associated risk of the studied mode, and the higher the priority in taking action, was. In this study, the IM line was segregated into four nodes corresponding to the main equipment parts. The team of authors participating in the preparation of the FMEA study consisted of two process operators and four safety consultants. A detailed walkthrough of the equipment and all processes within the injection moulding line was performed before finalizing the FMEA tables.

### 2.4. Control Banding

The FMEA approach is an advantageous tool for the identification, analysis, and discussion of physical- and machinery-related hazards and operator error-induced process safety events. The examination of nanomaterial hazards, on the other hand, requires a dedicated approach, seeing that FMEA does not possess the flexibility to include consideration of additional parameters that are integral to nanomaterial risk assessment (e.g., material characteristics, process details). The research field of nanosafety has displayed extended development and use of nanosafety assessment tools, often in web-based format, for aiding in nanomaterial process risk assessment [46]. These tools commonly rely on a control-banding approach [47] in which a user provides a set of basic information on the process performed (materials, process type, duration, etc.) and the tool assigns the material/process to a corresponding hazard and exposure band, forming the risk band. The risk band corresponds to a set of recommended control strategies, thus indicating the level and method of controls recommended. Control-banding methodologies have also been developed through nanosafety standards such as ISO/TS 12901-2:2014 [48]. However, web-based tools offer extended practicality and ease-of-use.

As mentioned in the Introduction, the imPURE approach involves nanomaterials being added to thermoplastics for introduction of antibacterial properties. Thus, the postrepurposing value chain introduces the stages of nanomaterial handling and incorporation in the thermoplastic masterbatch (compounding) before the injection moulding takes place. The handling of nanomaterials in powder, suspension, and solid nanocomposite physical states is commonly addressed as an exposure scenario in nanosafety tools [47]. For the purposes of risk assessment for this phase of the value chain, one of the most used control-banding tools, the Stoffenmanager Nano tool, was applied. Stoffenmanager Nano is a supportive control-banding tool for nanomaterial risk assessment developed by the TNO-Netherlands Organization for Applied Scientific Research (The Hague, The Netherlands) and Arbo Unie (Nijmegen, The Netherlands), and it is available as a web-based tool. Stoffenmanager Nano allocates five bands for hazard (A, B, C, D, E) and four bands for exposure (1, 2, 3, 4) on the basis of material and process characteristics, which are provided by the user. Task- and time-weighted exposure bands are derived based on the frequency and duration of a given process. The results from the hazard and exposure banding are combined in a risk matrix, which gives the risk priority band (1 = high priority, 2 = medium priority, or 3 = low priority) and thus the level of risk present [49]. The difference between the task- and time-weighted exposure bands lies in the function of the exposure algorithm of the Stoffenmanager Nano software. The task-weighted band informs about the exposure potential during a process described as a discrete event. On the other hand, the time-weighted band is based on a yearly risk prioritization, taking into account a 40 h/week work schedule as well as task intensity, frequency, and duration parameters input by the user [49].

## 3. Results

### 3.1. FMEA Risk Analysis

For the studied manufacturing pilot line, a total of 28 failure modes (FM) were identified by the operators and assigned to the respective nodes. Table 2 presents a short description for each FM as well as its source and potential effects. Most modes corresponded to the injection moulding and compounding nodes, since these nodes included the use of high-temperature/-pressure and heavy parts. Specifically, 12 failure modes were highlighted for injection moulding, 8 for the compounder, and 4 for the pelletizer and the oven. A wide variety of hazard types were identified within the manufacturing line, ranging from physical hazards (e.g., cuts, bruises, burns) to emission hazards (e.g., particles, fumes, nanomaterials) to hazardous events (e.g., potential eruption of pneumatic systems and electric shock).

Table 3 displays the ratings of the FMs and the groups of individuals who could be affected. The controls already in place for the mitigation of risks are also included. Severity and occurrence of risk related to inhalation hazards were lowered using general and local ventilation systems and respirators. Standard operating procedures (SOPs) and heavy-duty gloves helped mitigate the risk of mechanical hazard and burns.

### 3.2. Nanomaterial Risk Assessment

Exposure to engineered nanomaterials during compounding and that during injection moulding were classified as the highest-priority risks with *RPN*s of 48 and 32, respectively. For a more detailed assessment of the process steps involving ENMs, the activities were evaluated through the Stoffenmanager Nano tool. FM24 was described in the tool, given that it was the first level of handling of the nanomaterials, in the stage of nanopowder introduction to the compounder. Some notable inputs for the process description that were defined within the tool are as follows (Table 4):

- Ag nanoparticles were handled in dry powder form. Most commercial products of Ag nanoparticles that would be used within a repurposing-line context are available to purchase in dry powder form. It should be noted that the use of a suspension would lower the exposure risk but is impractical because of the requirement for incorporation in the thermoplastic.
- A low quantity of nanomaterials was required for each process task (approx. 10 g per 1 kg masterbatch).
- Small Ag nanoparticle size (<50 nm) was required for antimicrobial action.
- The masterbatch manufacturing process could have lasted several hours; however, the precise phase of nanoparticle handling and introduction was shorter in duration (up to 30 min per batch production).
- More than one employee was required to perform the task.

In order to showcase the risk assessment steps, three scenarios of increasing risk management efficiency were used to represent FM24 within the tool. The first one (Scenario A) indicated the inherent risk of the process, meaning the risk that would be present without any control measures being applied. The second (Scenario B) represented the risk levels after a conventional set of safety measures for emission control were applied (local exhaust ventilation (LEV) and filtering facepiece (FFP3) masks), while the third (Scenario C) represented an upgrade of the Scenario B safety system for further exposure potential reduction by containing the emission source:

- Scenario A—No controls applied (inherent process risk);
- Scenario B—Local exhaust ventilation and PPE applied;
- Scenario C—Local exhaust ventilation and source containment and PPE applied.

These scenarios were expressed only in the “Local control measures and personal protective equipment” tool module, since the process remained the same in all other aspects.

**Figure 3:**
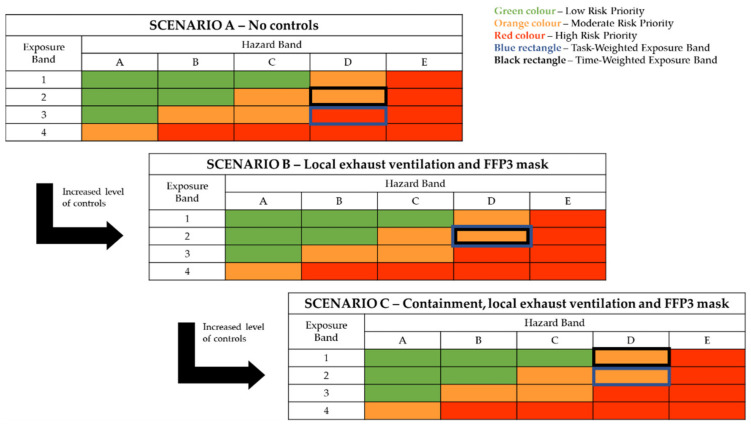
Control-banding classification of silver nanoparticles handling through Stoffenmanager Nano.

The risk assessment process led to the risk classification presented in Figure 3. The process was initially classified as high risk in terms of time- and task-weighed exposure if no controls were applied. The application of LEV and PPE (FFP3 masks) controls reduced the task-weighed exposure to more controllable, moderate risk levels. Further risk reduction (in terms of time-weighed exposure) was achieved by complementing LEV and the use of FFP3 masks with enclosure of the source. The use of local exhaust ventilation and particulate respirators is a fundamental safety recommendation for the containment of nanomaterial exposures during extrusion/compounding processes involving nanomaterial additives, according to the United States National Institute for Occupational Safety and Health (NIOSH) [50]. The NIOSH guidelines also specified setup parameters for the LEV system, such as placing the exhaust pickup of the LEV as close to the emission source as possible, as well as air hood capture air velocity requirements of at least 0.5 m/s at the specific point of expected particle release. This set of guidelines constitutes an effective strategy for exposure mitigation. However, the inherent nanomaterial hazard potential is not affected. Reducing the risk lower than that of Scenario C according to the Stoffenmanager Nano scoring system would be possible only by reducing the hazard of the material, which would require application of a hazard/toxicity reduction technique, such as increasing the primary size of the nanoparticles [51] or applying a different capping agent [52]. However, it should be noted that such an alteration in the primary nanomaterial additive may be disruptive to the effectiveness of the material as an antimicrobial agent in the present application. Consistently with the tool output, in an exposure assessment study of nanocomposite compounding processes, Tsai et al., confirmed that collective use of enclosure of the emission source and LEV led to a significant reduction in exposure to released nanoparticles [53]. Interestingly, the authors noted that another type of controls, those of administrative nature (e.g., cleaning practices, dust removal from equipment), may have resulted in substantial exposure reduction. However, these types of controls are complicated to feature accurately in control-banding methodologies or tools, and their impact cannot be quantified easily within a banding approach.

## 4. Discussion

### 4.1. Prioritization of the Failures and Associated Risks

Failure modes were prioritized based on their *RPN*s. For the 28 failure modes identified, *RPN* values ranged from 8 to 48, with a median of 12. For the purposes of this study, seven failure modes were selected for further analysis, representing the highest threats (Table 5) and corresponding to the fourth quartile of *RPN* values, with values ranging between 24 and 48. Concerning failure modes FM12 and FM24, part manufacturing and end-of-life steps of polymer materials can lead to the emission of ultrafine particles (UFP), which are airborne particles with diameters of less than 100 nm. Injection moulding of polymer materials has not been thoroughly studied in regard to the emission potential of UFPs, which can adversely affect operators’ health. However, several studies, including ones by Theriault et al. [54] and Boonruksa et al. [35], have indicated the emission of UFPs during the IM procedure in neat and additivated polypropylene (PP) and polycarbonate (PC) polymer materials. Based on the findings of Theriault et al., airborne particulate emissions can differ greatly (i.e., >30%) between feedstocks because of the potential oxidative degradation of some polymers during IM leading to high emissions [54]. The required safety control protocols as mitigating actions against the particulate emissions in IM manufacturing lines have not yet been thoroughly defined in the literature. Furthermore, extensive scientific research investigating UFP emissions of polymer materials, including ABS, has already been conducted in the similar field of 3D printing, confirming UFP emissions during thermoplastic material—and specifically ABS—manufacturing procedures [55]. In 3D printers, the issue of UFP emissions can be dealt by enclosing the printer and applying an air recirculation system with high-efficiency particulate absorbing (HEPA) filtration. Administrative controls such as remote monitoring of the process to avoid exposure also apply. However, these methods are not directly applicable to IM or compounding processes because of the larger size of the equipment compared with most FFF 3D printers. Custom fabricated partial enclosures and containment of the emission source are more compatible as emission control strategies for IM and compounding process setups. These exposure mitigation aspects are important, since the health hazards of UFP have been acknowledged in the literature. It has been demonstrated that UFPs can cause a variety of adverse health effects due to high respiratory system penetration [27]. They can penetrate the cellular membrane and lead to the production of ROS. UFPs’ induced cytotoxicity is originated by the developed oxidative stress causing DNA damage, oxidation and denaturation of proteins and enzymes, and disruption of mitochondria, leading to cell apoptosis as well as greater adverse health effects such as inflammation, chronic respiratory illnesses, and cancer [56]. Consequently, adverse health effects due to UFP emissions of ABS material during the IM procedure must be seriously taken into account. Incorporating AgNPs in a manufactured product increases the overall risk of the product’s manufacturing and end-of-life stages by introducing the threat of exposure to polymer/ENM aggregates during the injection moulding line and shredding activities. Exposure to free silver nanoparticles (without polymer matrix) is also possible during the initial handling of the ENMs [48]. In addition to the innate hazards due to the small particle size, in vitro and in vivo studies have showcased that exposure to AgNPs can lead to cytotoxicity, ROS generation, and DNA damage. Primary exposure can occur through all main routes (respiratory, oral, skin), leading to local effects on the corresponding pathways. Ag particles and ions can also reach several organs through translocation, potential even penetrating the blood–brain barrier [57].

Some failure modes were connected to specific equipment and infrastructure parts of the line (e.g., pressure valve failure for FM11 and FM23). These events were closely connected to the equipment failure rates, most commonly defined in safety studies through failure rate libraries [58]. The further study of these failure modes would require the introduction of more quantitative data concerning probability of occurrence and could be conducted through a methodology such as probability bow-tie analysis, although such quantitative risk assessment methodologies are more common in plants of larger scale [59]. Extensive experience in risk assessments based on reliability data of infrastructure elements has been established in the oil and gas industry [60] because of the large scale of the infrastructure employed. Such aspects were not present in the current study, where quite a few process tasks were performed manually by the operators and the production was performed on a lab/pilot scale. Implementation of probability-based approaches would require the collection of failure data on these specific safety compartments from multiple sources with a view to developing the relevant failure rate libraries for the technologies in question. An inspection and maintenance program for the compressor and related pressure valves [61] is important to reduce the residual risk as much as reasonably practicable. Moreover, concerning FM23, appropriate safety measures for protection against electrocution (electric shock) are compulsory due to the potential short-circuit caused by water spillages. Specifically, for the compounder, the cooling water bath for the extruded material should not be close to exposed cables, and all electrical equipment of the pilot line must be properly insulated. Adjustable guards on the sides of the water tank bath and spill absorbent pads can further reduce the risk.

Regarding FM1, Rafat et al. [62] reported an injection moulding machine accident in which a worker was fatally injured while attempting to remove hardened plastic from the mould of a hydraulic, horizontal thermoplastic injection moulding machine. Despite the scale of the injection moulding machine being much larger than that of the machine we studied, this incident highlights the seriousness FM1 can acquire, as in the pilot line where the accident was reported, several mechanical, electrical, and hydraulic safety devices had already been installed.

The Canadian Institut de Recherche Robert-Sauvé en Santé et en Sécurité du Travail (IRSST) has conducted extensive work related to process safety in injection moulding and has compiled data related to safety incident reporting for large-scale injection moulding processes [63]. In [63], a safety incident was documented in which the release of gases from ABS thermal degradation combined with an ignition source and led to the outburst of fire, inflicting burns to an employee. For small-scale IM processes and low quantities of material being processed, gases released could not lead to concerningly high concentrations given that proper fume extraction controls were in place. This synergistic hazard potential (fume inhalation/ignition potential) concerning FM4 and FM19, which relate to the emission of ABS fumes, may emerge in the case of continuous operation without any ventilation or extraction equipment in use. Nevertheless, the documentation of such incidents or near-miss events is very important to establish, as also encouraged by the European Commission’s Joint Research Centre (JRC) [64]. This is especially relevant in the currently developing nanocomposite IM industry, as it may serve as a basis for developing more robust safety systems in view of the generated knowledge on potential safety incidents.

Regarding FM4 and FM19, it should be noted that the potential health hazard of the fumes emitted is directly related to the particular species of agent released. Specific studies dedicated to volatile organic compound (VOC) release analysis are not available for IM to the best of our knowledge. However, within this context, the HSE has highlighted the potential for release of several hazardous fumes from ABS plastic thermal processing while also emphasizing that heating temperature is a critical determinant of fume production [65]. Substantial work related to the release of fumes from ABS material under thermal processing conditions has been undertaken in another field of thermoplastic material manufacturing, that of 3D printing. ABS is one of the most common filament feedstock materials for FFF 3D printing [66], and thus, several studies have been conducted to investigate its emissions during the process, consistently reporting that styrene is commonly emitted [67]. Control of exposure to VOCs emitted from 3D printing can be facilitated via the installation of an extraction system in which the exhaust is filtrated with activated carbon filters, and by principle, this concept could be adapted to the processes of the present work. Further discussing FM4 and FM19, the processing (injection moulding) of ABS to final products requires elevated temperatures. This induces partial breakdown and decomposition of the polymer, giving rise to a complex mixture of airborne compounds and fumes (VOCs) that may contaminate the workroom air. Many of these products are potentially toxic, e.g., styrene, acrylonitrile, butadiene, bisphenols, and other organic nitriles. Human exposure to fumes and VOCs due to ABS degradation has been a topic of concern because of the mutagenic, genotoxic, and carcinogenic potential of their emitted monomers. Furthermore, several ABS-related VOCs are suspected to be potent central nervous system toxicants [68].

Zitting et al. [69] exposed rats to fumes from thermal degradation of an ABS material in order to examine their biochemical effects on the brain, kidneys, liver, and lungs. They found that long-term (2-week) exposure to ABS degradation fumes decreased hepatic, lung, kidney, and cerebral glutathione (GSH) concentration levels. Glutathione plays an important biological role in preventing damage to important cellular components caused by ROS such as free radicals. Thus, the decrease in the GSH concentration suggests that serious hypoxic and peroxidation mechanisms are involved in the reaction to the exposure. Moreover, Dematteo et al. [70] investigated occupational exposure of female workers to chemicals used in the plastics industry. They concluded that exposure to fumes and VOCs, including several aforementioned compounds from ABS processing and thermal degradation (such as styrene, acrylonitrile, and butadiene), may contribute to the development of breast cancer (mammary carcinogen action) and disrupt normal function of reproductive and endocrine systems. A confirmation of these came from scientists at the Silent Spring Institute in Massachusetts [71], who listed the monomers acrylonitrile, styrene and 1,3-butadiene in their comprehensive database of substances shown to cause mammary gland tumours in animals. Acrylonitrile has been linked to genital abnormalities in children born to exposed mothers and may also induce endocrine disrupting effects [72], while styrene, in addition to being a possible carcinogen, has been identified as an endocrine disruptor [73]. Furthermore, 1,3-butadiene has been shown to induce mammary gland tumours in rats and was classified by the International Agency for Research on Cancer (IARC) as a Group 2A carcinogen [74].

### 4.2. Hierarchy of Protective Measures for Repurposed Injection Moulding Units

Based on the Hierarchy of Controls scheme, as described in ISO 45001:2018 Occupational health and safety management systems—Requirements with guidance for use [75], a multitude of engineering controls, administrative actions, and case-specific personal protective equipment can be applied for each of the seven failure modes representing the highest threats as prioritized in Section 4.1. For failure modes regarding inhalation of ENMs, UFPs, and hazardous fumes (FM24, FM12, FM4, and FM19), several recommendations concerning the application of specific engineering controls can be provided in order to help in exposure mitigation. The processes of the pilot line (injection moulding and compounder) must be performed, if possible, either within a custom fabricated ventilated enclosure to capture and remove the emitted particles and toxic fumes from the workroom air, or within a partially enclosed ventilated setup. Moreover, the use of LEV systems such as wide canopy hoods and arm hoods for emission removal and exposure mitigation is recommended. In case local ventilation is applied, the existing hoods should be shaped in order to enclose the source as much as possible and be as close to the emission source as possible. Furthermore, it is highly recommended to use a HEPA filter class H14 for the outflow filtration of the exhaust air and harmonize the area with general dilution ventilation requirements for laboratories (10 air changes per hour, ACH) [76].

Recommended best practices and administrative controls, critical in exposure mitigation and consequently in operators’ safety, include proper signage of the workplace area about nanohazards, isolation of the workplace area from non-nanoparticle-related activities, allowing access to the area to authorized and trained persons only, and performing regular maintenance testing of the extraction ventilation equipment. Concerning PPE, the use of a FFP3 respirator for the whole duration of the process is considered necessary. An upgrade to full-/half-face respirators with P3 particulate filters and appropriate filters for organic toxic fumes and volatile organic compounds must be considered depending on emitted UFPs, ENMs, and VOCs. In addition, according to the EC recommendations, operators and workers involved in nanomaterial processes should be face-fit tested for the respirators they use as personal protective equipment via a quantitative fit testing procedure. Quantitative respirator fit testing ensures that maximum protection is offered by the respirators, particularly against nanoparticles, the high mobility and low mass of which renders them capable of passing through microscopic gaps left by an improperly fitting respirator [77]. Double nitrile, latex, or neoprene gloves of >100 μm thickness and <1.5 acceptable quality limit (AQL) index are also necessary, as well as safety goggles for ocular protection. Finally, a spill containment protocol should be in place in case of an emergency.

Concerning FM11 (eruption), administrative controls, such as a periodic inspection and maintenance program for the compressor, tubing, and related pressure valves, are important in order to reduce risk and prevent a possible eruption. In the case of FM23 (electric shock), all electrical equipment of the pilot line must be properly insulated by design. Furthermore, the cooling water bath for the extruded material should not be close to exposed cables and sockets. In this way, protection is ensured against electrocution due to a short-circuit caused by water spillages. In addition, engineering controls and appropriate safety measures, such as spill-absorbent pads beneath the equipment/machinery and adjustable guards on the sides of the water tank bath, further reduce the risk. The use of special insulated gloves should also be taken into consideration.

For the failure mode concerning cuts, bruises, and injuries (FM1), administrative controls, such as training of personnel for proper use of the machinery/equipment, labelling of the equipment for any physical hazards arising during the operation of the pilot line, and developing written operating procedures for the convenience of the operators, can reduce the existing risk. In addition, concerning PPE, operators must be equipped with cut- and heat-resistant work gloves (comfort-grip gloves) when the equipment of the pilot line is under load, as well as with mandatory safety goggles for ocular protection. Further discussing FM1, the intake area of the feeding system’s opening should be protected by design by a fixed hopper or another fixed guard.

The other failure modes, although presenting lower *RPN* scores in Table 2 and Table 3 due to easier detection or lower potential impact and probability, require their own sets of controls to reduce residual risk as much as reasonably practicable. The lowest *RPN* was presented for the electrical-type hazards related to the use of the equipment (FM8, FM21, and FM28), given that equipment was CE-marked and appropriately installed and set up. Dust emissions that may be produced from pelletizer use (FM14, FM15) and the last step of the injection process (FM5, FM6) can be adequately controlled through the emission control and safety measures described for nanoparticle and nanomaterial exposure (FM12, FM24), such as ventilation, respiratory protection, and safety glasses. Various parts of the production line entail high-temperature hazards (FM2, FM3, FM17, FM18, FM25, and FM26), which can be mitigated through the use of heat-resistant gloves for any necessary direct interference with hot parts of the equipment as well as proper risk perception and following the standard operating procedures. It is noted that the definition of and strict adherence to SOPs constitute a fundamental safety measure for many of the FMs, indicating the importance of administrative controls within a comprehensive safety system. FM9, which displayed an *RPN* score of 15, concerned the possibility of hearing damage as a result of the compressor use, which can be controlled through placing of the noise-inducing equipment in a remote room. Isolation of the noise sources can be applied on a priority basis as a noise exposure control, and the application of engineering controls such as acoustic screens and barriers is also recommended before personal protection is implemented. For occasional attendance in noisy areas, a noise refuge may be considered, which consists of an employee-occupied acoustic enclosure aimed to keep noise out [78]. Given that noise presented safety issues with only one of the elements of the present process setup (the compressor), administrative controls such as job rotation could be considered to avoid the costlier engineering interventions. However, if work in this particular area is needed and no other engineering or administrative measures can be applied, the use of hearing protection (earmuffs or earplugs) is critical.

### 4.3. Limitations of Study and Potential for Further Research

The intrinsic characteristics of the methodologies applied in the present study entailed several limitations. It should be noted that exposure, as described within the tool input in Section 3.2, refers to exposure to airborne nanomaterials from the phase of nanopowder handling and addition within the masterbatch. However, exposures during the stages of thermal and mechanical stress of the material during the main processes of compounding and injection moulding phases should not be neglected. The exposure scenario involving the release of nanomaterials from a nanocomposite is outside of the scope of the Stoffenmanager Nano tool, since nanocomposites are not examined via this tool [49]. An alternative approach or tool should be used to assess the exposure risk involved in this scenario. In general, control-banding tools focus on the synthesis and handling phases. The various types of nanocomposite processing activities have not been extensively integrated into most nanosafety tools as of yet [47]. More specifically, the release of nanomaterials during thermal processing of nanocomposites is not included as a separate exposure scenario in control-banding tools or methodologies [48,79,80]. The most closely corresponding exposure scenarios that refer to nanocomposites are “mechanical processing” of nanocomposites (e.g., machining, drilling). If this exposure scenario were to be a feature of a nanosafety tool, it could be examined in parallel with powder handling. This would enable further study of FM12, which described the potential for inhalation of emitted nanomaterials from the IM process. Since the risk band and safety recommendations would derive from this analysis, further optimization of the safety system would be supported based on the developed risk priority between the processes.

On-site exposure assessments based on airborne (nano)particle release measurements constitute a robust method to quantify the potential occupational risks posed by handling nanomaterials. These exposure studies often involve several visits and process walkthroughs in the workplace under investigation, using a diverse set of instruments to get airborne particle concentration readings or workplace air samples, and they may use tiered or “step-wise” approaches [81]. Supporting the present study with an occupational exposure measurement campaign would most certainly benefit the accuracy of the assessment results while also helping to study the more challenging failure mode (FM12) related to the releases from nanocomposites. However, it is worth noting that these assessments require specialized equipment, resources, and know-how on specific topics such as using the equipment, performing reliable data gathering through specific methodologies, and interpreting the results [82]. These aspects accentuate two of the major barriers of the repurposing process, the financial requirements and the skill gap. Along these lines, for the part of the assessment concerning conventional hazards, a more complex approach such as a hazard and operability study (HAZOP) would benefit the assessment in terms of output accuracy [83]. Such a method would require the definition of distinct nodes of the repurposed line, similarly to the scheme that was set up in the FMEA examination of this study. However, a HAZOP would also require the segregation of the process into sequential steps or subsystems (e.g., filling the barrel, demoulding, cleaning the barrel, etc.) in order to examine each step’s deviations through the HAZOP’s structured guidewords (e.g., LESS input material, EARLY demoulding, AFTER cooling). Similarly to the exposure measurements, although producing results of higher detail, a HAZOP would require greater allocation of time, resources, and workforce.

Seeing that one of the primary alterations in the process was the introduction of nanomaterials, aside from safety aspects, the environmental impact of such a process modification would be important to study as well [84]. This would require a life cycle assessment (LCA) study [85], which could reveal the potential additional burdens or benefits introduced by this novel repurposed product life cycle. Especially for silver nanoparticles, concerns over their toxicity to various organisms after release into the environment has been expressed, particularly because of the expanded use of AgNP-enabled commercial products and the resulting high potential for aquatic environment release [86]. The impact of AgNPs on the environment and their potential ecotoxicity are closely connected to their physicochemical characteristics, and thus, work has been performed to mitigate their ecotoxicity through structural modifications [87] A comprehensive exposure control strategy, along the lines of the present work, could be supported by such interventions to reduce environmental hazard and work towards all-round sustainable AgNP solutions. The life cycle impact of AgNP-enabled items has been examined through LCA studies for other types of products, such as socks [88]. The authors of [88] noted that the life cycle impacts may be closely connected to the synthesis route of silver nanoparticles and stressed that impacts may become more significant if global scaling up of the production and use of silver nanoparticles were realized. An LCA-based comparison of pre- and postrepurposing environmental impacts could serve as a vital screening tool for assessing the environmental aspects of the repurposing approach and potentially identify ways to mitigate the environmental footprint.

## 5. Conclusions

This study presents an innovative safety-oriented FMEA regarding a repurposed injection moulding manufacturing line as part of the imPURE project. A low concentration of silver (Ag) nanoparticles was added in a polymer matrix because of their antimicrobial properties. Twenty-eight failure modes were identified in the four nodes of the repurposed manufacturing process (injection moulding, compounder, pelletizer, and oven), and seven of these were selected for further analysis. These seven represented the riskiest identified threats, including cuts, bruises, and injuries during operation; electric shock; eruption of high-pressure vessels; hearing damage due to high noise levels; inhalation of hazardous fumes; and exposure to ENMs. The FM concerning inhalation of UFPs and ENMs presented the highest *RPN* because of high respiratory system penetration leading to adverse health effects. For a more detailed assessment of the process steps involving ENMs, the activities were evaluated through the Stoffenmanager Nano tool via a control-banding classification of AgNP handling, showing that the use of LEV, source containment, and FFP3 respirators would lower the task- and time-weighted exposure to moderate risk bands. Based on the Hierarchy of Controls scheme, a multitude of engineering controls, best practices, administrative actions, and case-specific PPE were proposed for each of the emerged threats. Administrative actions such as SOPs, signage, and periodic maintenance would mitigate the risks from eruption, cuts, and bruises, while the appropriate ventilation flow rate, LEV and HEPA filtration, and FFP3 respirators and P3 filters would minimize UFP, ENM and VOC exposure. This work provides the necessary guidelines for risk assessment and occupational health and safety management of repurposed industrial production lines of critical medical devices during the pandemic era. Extended studies focusing on the safety and sustainability aspects of such repurposing setups would be valuable for establishing and optimizing the long-term viability of manufacturing line repurposing approaches.

## Figures and Tables

**Table 1:** Evaluation of the three failure mode and effect analysis (FMEA) factors.

Level	Severity (*S*)	Occurrence (*O*)	Detection (*D*)
1	Negligible	Almost impossible	Almost certain
2	Minor	Low	High
3	Serious	Moderate	Moderate
4	Major	High	Low
5	Fatal	Almost certain	Almost impossible

**Table 2:** Failure modes per piece of equipment of the repurposed injection moulding line.

Failure Modes	Node	Threat	Source	Description
FM1	Injection Moulding	Cuts, bruises, injuries	Moving mechanical parts	Injuries caused by moulds dismantling tooling
FM2		Burns	Hot surfaces	Exposed hot surfaces
FM3		Burns	Heated material	Hot specimens from the mould
FM4		Inhalation of hazardous fumes		Melted or heated polymer
FM5		Eye irritation/damage	Dust	Dust during the final step of the injection process (relaxation)
FM6		Respiratory irritation/damage		
FM7		Explosion	High pressure	Operation at max 16 bar through a pneumatic system
FM8		Electric shock	Electrical hazards	Equipment was connected to the power grid
FM9		Hearing damage	Noise	Mainly due to the compressor (>100 dB). Disturbing periodic noise from the vent
FM10		Strain injury, injury from falling object	Lifting of heavy parts	Moulds for IM
FM11		Eruption	Compressor operation and emptying	High-pressure 90 L vessel on compressor and tubing below the flooring at approx. 15 bars
FM12		Inhalation of/dermal contact with ENMs	Handling of ENMs	Nanocomposite masterbatch production
FM13	Pelletizer	Cuts, bruises, injuries	Moving mechanical parts	Cuts during pelletizer operation (blades at high rotation speeds)
FM14		Eye irritation/damage	Dust	Mainly from the pelletizer
FM15		Respiratory irritation/damage		Mainly from the pelletizer
FM16		Electric shock	Electrical hazards	Equipment connected to the power grid
FM17	Compounder	Burns	Hot surfaces	Exposed hot surfaces
FM18		Burns	Heated material	Melted polymer from the compounder
FM19		Inhalation of hazardous fumes		Due to melted or heated polymer
FM20		Explosion	High pressure	Compounder can reach up to 80 bars
FM21		Electrical shock	Electrical hazards	Equipment connected to the power grid
FM22		Strain injury, injury from falling object	Lifting of heavy parts	Feeders for compounder
FM23		Electric shock	Water spillage	Cooling water bath for the extruded material (close to cables, sockets, etc.)
FM24		Inhalation of/dermal contact with ENMs	Handling of ENMs	Nanoparticles inserted in the compounder through a gravimetric feeder in dry form
FM25	Oven	Burns	Hot surfaces	Exposed hot surfaces
FM26		Burns	Heated material	Hot pellets dried in the oven
FM27		Inhalation of hazardous fumes		Due to the heated polymer
FM28		Electric shock	Electrical hazards	Equipment connected to the power grid

**Table 3:** Failure mode and effect analysis (FMEA) of the repurposed injection moulding line.

Failure Modes	Affected Groups	Existing Controls	*S*	*O*	*D*	*RPN*
FM1	O	Standard operating procedure (SOP), heavy-duty gloves, pelletizer interlock	2	4	3	24
FM2	O	SOP, heavy-duty gloves, safety cover	3	4	1	12
FM3	O	SOP, lab coat, heavy-duty gloves, closed shoes	3	4	1	12
FM4	O, B	Arm hood, central ventilation system, respirators	3	4	2	24
FM5	O, B	Arm hood, central ventilation system, safety glasses	2	2	3	12
FM6	O, B	Arm hood, central ventilation system, respirators	2	2	3	12
FM7	O, B	Fire extinguisher, fire plan, safety glasses, pressure indicators on the compressor and IM machine, pressure sensors on the compounder with emergency shutdown	5	1	2	10
FM8	O	Lightning arrester in the switchboard, current relay, extra grounding, power safety for the operation bench	4	1	2	8
FM9	O, B	Isolation of compressor to a separate room	3	5	1	15
FM10	O, B	SOP, closed shoes	3	4	1	12
FM11	O, B	SOP, pressure valve and indicator	5	3	2	30
FM12	O, B	Isolated inlet system: material is filled in the feeder under a fume hood and then placed on the extruder feeder. Arm hood above the feeder. PPEs (face mask, gloves, lab coat, shoe covering, hair covering)	2	4	4	32
FM13	O	SOP, heavy-duty gloves, pelletizer interlock	2	3	2	12
FM14	O, B	Arm hood, central ventilation system, safety glasses	2	2	3	12
FM15	O, B	Arm hood, central ventilation system, respirators	2	2	3	12
FM16	O	Lightning arrester in the switchboard, current relay, extra grounding, power safety for the operation bench	4	1	2	8
FM17	O	SOP, safety cover, heavy-duty gloves	3	4	1	12
FM18	O	SOP, lab coat, heavy-duty gloves, closed shoes	3	4	1	12
FM19	O, B	Arm hood, central ventilation system, respirators	3	4	2	24
FM20	O, B	Fire extinguisher, fire plan, safety glasses, pressure indicators on the compressor and the IM machine, pressure sensors on the compounder with emergency shutdown	5	1	2	10
FM21	O	Lightning arrester in the switchboard, current relay, extra grounding, power safety for the operation bench	4	1	2	8
FM22	O, B	SOP, closed shoes	3	4	1	12
FM23	O	SOP, release valve on the bottom of the bath	5	3	2	30
FM24	O, B	Isolated inlet system: material is filled in the feeder under a fume hood and then placed on the extruder feeder. Arm hood above the feeder. PPEs (face mask, gloves, lab coat, shoes covering, hair covering). Use of liquid pump to insert the particles in suspension form	3	4	4	48
FM25	O	SOP, heavy-duty gloves, safety cover	3	4	1	12
FM26	O	SOP, lab coat, heavy-duty gloves, closed shoes	3	4	1	12
FM27	O, B	Arm hood, central ventilation system, respirators	3	2	2	12
FM28	O	Lightning arrester in the switchboard, current relay, extra grounding, power safety for the operation bench	4	1	2	8

O: operators; B: background personnel.

**Table 4:** Input parameters in the Stoffenmanager Nano for silver nanoparticle hazard assessment.

Stoffenmanager Nano Input Fields		Parameters Input within Tool
Process Type		Handling of Bulk Aggregated/Agglomerated Powders
Product Characteristics	Dustiness	Unknown
	Moisture content	Dry product
	Concentration of nano component	100%
	Does it contain one of the following OECD components?	Ag (nanosilver)
	Is the primary particle diameter larger than 50 nm?	No
Handling/Process	Task characterization	Handling of products in small amounts (up to 100 g)
	Task duration	30–120 min a day
	Task frequency	2–3 days a week
	Task performed within breathing zone of employee	Yes
Working Area	Working room being cleaned daily;	Yes
	inspections and maintenance of machines/ancillary equipment being done at least monthly	Yes
	Working room volume	100–1000 m^3^
	Ventilation in working room	Mechanical or natural ventilation
Local Control Measures and Personal Protective Equipment	Local control measures	Scenario A—No controls Scenario B—Local exhaust ventilation Scenario C—Containment of the source with local exhaust ventilation
	Personal protective equipment	Scenario A—No controls Scenario B—FFP3 mask Scenario C—FFP3 mask

**Table 5:** Ratings for highest threat failure modes.

Priority Number	Failure Modes	Affected Groups	*S*	*O*	*D*	*RPN*
1	FM24	O, B	3	4	4	48
2	FM12	O, B	2	4	4	32
3	FM11	O, B	5	3	2	30
4	FM23	O	5	3	2	30
5	FM1	O	2	4	3	24
6	FM4	O, B	3	4	2	24
7	FM19	O, B	3	4	2	24

O: operators; B: background personnel.

## Data Availability

Data is contained within the article.
